# The impact of estradiol on serotonin, glutamate, and dopamine systems

**DOI:** 10.3389/fnins.2024.1348551

**Published:** 2024-03-22

**Authors:** Peyton Christine Bendis, Sydney Zimmerman, Anna Onisiforou, Panos Zanos, Polymnia Georgiou

**Affiliations:** ^1^Psychoneuroendocrinology Laboratory, Department of Psychology, University of Wisconsin Milwaukee, Milwaukee, WI, United States; ^2^Translational Neuropharmacology Laboratory, Department of Psychology, University of Cyprus, Nicosia, Cyprus; ^3^Laboratory of Epigenetics and Gene Regulation, Department of Biological Sciences, University of Cyprus, Nicosia, Cyprus

**Keywords:** estradiol, estrogen, estrogen receptors, dopamine, serotonin, glutamate

## Abstract

Estradiol, the most potent and prevalent member of the estrogen class of steroid hormones and is expressed in both sexes. Functioning as a neuroactive steroid, it plays a crucial role in modulating neurotransmitter systems affecting neuronal circuits and brain functions including learning and memory, reward and sexual behaviors. These neurotransmitter systems encompass the serotonergic, dopaminergic, and glutamatergic signaling pathways. Consequently, this review examines the pivotal role of estradiol and its receptors in the regulation of these neurotransmitter systems in the brain. Through a comprehensive analysis of current literature, we investigate the multifaceted effects of estradiol on key neurotransmitter signaling systems, namely serotonin, dopamine, and glutamate. Findings from rodent models illuminate the impact of hormone manipulations, such as gonadectomy, on the regulation of neuronal brain circuits, providing valuable insights into the connection between hormonal fluctuations and neurotransmitter regulation. Estradiol exerts its effects by binding to three estrogen receptors: estrogen receptor alpha (ERα), estrogen receptor beta (ERβ), and G protein-coupled receptor (GPER). Thus, this review explores the promising outcomes observed with estradiol and estrogen receptor agonists administration in both gonadectomized and/or genetically knockout rodents, suggesting potential therapeutic avenues. Despite limited human studies on this topic, the findings underscore the significance of translational research in bridging the gap between preclinical findings and clinical applications. This approach offers valuable insights into the complex relationship between estradiol and neurotransmitter systems. The integration of evidence from neurotransmitter systems and receptor-specific effects not only enhances our understanding of the neurobiological basis of physiological brain functioning but also provides a comprehensive framework for the understanding of possible pathophysiological mechanisms resulting to disease states. By unraveling the complexities of estradiol’s impact on neurotransmitter regulation, this review contributes to advancing the field and lays the groundwork for future research aimed at refining understanding of the relationship between estradiol and neuronal circuits as well as their involvement in brain disorders.

## 1 Introduction

17β-estradiol (E2) is a naturally occurring steroid hormone synthesized by the ovaries in females or produced through the aromatization of testosterone in males (Cooke et al., 2017; Hariri and Rehman, 2023). While it plays a crucial role in maintaining the reproductive system, its influence extends beyond, encompassing functions such as modulation of the cardiovascular system, mood, memory, and cognition (Russell et al., 2019; Ueda et al., 2020; Hwang et al., 2021). Traditionally recognized as a sex hormone with daily or monthly fluctuations in females, including rodents and humans, E2 is also widely distributed in the male brain, derived from the conversion of testosterone through aromatase enzymes (MacLusky et al., 1987; Sharpe, 1998; Simpson et al., 2000; Danielson, 2002). It worth noting that in addition to the role of E2 in males and females, it plays an important role in intersex individuals. Specifically, excess estrogen production or sensitivity can influence the development of female secondary sex characteristics in people with XY chromosomes (Gillies and McArthur, 2010; Wisniewski, 2012). Conversely, individuals with XX chromosomes and undervirilization (incomplete masculinization) might have lower estrogen levels, impacting their pubertal development and potentially requiring estrogen replacement therapy later (Gillies and McArthur, 2010; Wisniewski, 2012). It is crucial to remember that intersex is a diverse spectrum, and the relationship between estrogen and individual presentations is highly nuanced.

E2 exerts its effects by binding to estrogen receptors α or β (ER alpha and beta) and G protein-coupled estrogen receptor (GPER). Estrogen receptors can function as transcription factors, binding to the estrogen response element (ERE) to initiate transcription, or act as membrane receptors. Specifically, ERα and ERβ, as transcription factors, target and regulate distinct genes. Membrane bound estrogen receptors (mERs) achieve the same results acting rapidly through kinases and signaling pathways. Similar to mERs, GPER utilizes signaling pathways and protein kinases to achieve transcription of E2-regulated genes. Although activated by estrogens (Estrone—E1, 17β-estradiol—E2, Estriol—E3, Estetrol—E4), with E2 being the most potent ligand, these receptors can also be activated independently without ligand binding through mechanisms such as activation by phosphorylation of kinases or transcription factor cross-talk. Both receptors share some target genes, yet exhibit unique transcriptional effects in different cell types, influenced by ligands and binding sites (Zhao et al., 2008).

ERα is predominantly expressed in breast tissue, uterus, ovaries (thecal cells), prostate stroma, and the liver, whereas ERβ is most highly expressed in the ovaries (granulosa cells), lungs, prostate epithelium, colon, and the immune system (Hewitt et al., 2000; Dahlman-Wright et al., 2006; Heldring et al., 2007; Paterni et al., 2014; Bondesson et al., 2015). GPER is expressed in a variety of tissues including reproductive tissues, adrenal, kidney, pancreatic islets, liver, skeletal muscle endothelium, breast, intestine, and inflammatory cells (Carmeci et al., 1997; Haas et al., 2007; Wang et al., 2007; Dennis et al., 2009; Hazell et al., 2009; Prossnitz and Barton, 2011). Nevertheless, all three receptors are present in the cardiovascular system, nervous system, and adipose tissues (Dahlman-Wright et al., 2006; Heldring et al., 2007; Prossnitz and Barton, 2011; Paterni et al., 2014). In females, E2 is predominantly localized in the ovaries, adrenal glands, and fat tissue, while males synthesize E2 in the testes, adrenal and pituitary glands. These sex-specific differences suggest that E2 may lead to significant variations in physical and emotional responses between males and females. Ongoing research aims to deepen our understanding of which receptor subtype should be targeted for the treatment of various pathological disorders.

The regulation of gene transcription by ER involves two mechanisms: classical (genomic-slow) and nonclassical (non-genomic-fast). In the classical mechanism, E2 binds to inactive ERs upon entering the cell. Steroid hormones, being nonpolar molecules, enable E2 to diffuse across the cell membrane and enter the cell (Oren et al., 2004). The inactive ER is associated with heat shock proteins, HSP70 and HSP90. It undergoes dissociation from the heat shock protein upon binding with E2, thereby activating the ER. ER dimerization follows, where two activated ERs ionically bind to each other, forming either heterodimers (ERα bound to ERβ) or homodimers (ERα bound to ERα or ERβ bound to ERβ). Dimerization facilitates the entry of ERs into the nucleus, targeting the ERE within DNA. Successful transcription of E2-regulated genes requires the recruitment of basal transcriptional machinery and coregulator proteins CoA and CoR (Björnström and Sjöberg, 2005; Le Dily and Beato, 2018).

The majority of research focuses on the genomic mechanisms of ERs, however, exploration of the non-genomic mechanisms may lead to a better understanding of the rapid effects of E2 and ERs. Srivastava, et al., have shown an abundance of extranuclear ERα and ERβ immunoreactivity within the adult mouse hippocampus suggesting the presence of mERs. Alongside the localization of ERs, they noted the influence of the estrus cycle on receptor expression. ERα showed greater expression during diestrus and in males compared to ERβ where expression was higher during estrus and diestrus and mild during proestrus and in males. During high periods of estrogen, such as during proestrus or the luteal phase, nuclear ER expression decreases (Mitterling et al., 2010) indicating another mechanism responsible for E2-mediated transcription besides nuclear ERs. When utilizing the non-genomic mechanism, ER achieves its transcriptional effects without binding to DNA. This can occur through a process called transcriptional cross-talk, where signaling happens via protein–protein interaction between receptors (Göttlicher et al., 1998). Another way to achieve transcription through non-genomic mechanisms is through extracellular E2 interacting with membrane bound GPER. GPER activation can lead to mobilization of intracellular calcium ions, cyclic adenosine 5′-monophosphate (cAMP) production, activation of protein and lipid kinases, and activation of the MAPK and PI3K signaling pathways (DeLeon et al., 2020). Once cAMP is produced, it can enter the PI3K signaling pathway to activate protein kinase cascades. These cascades phosphorylate and activate transcription factors, resulting in the transcription of E2-related genes. Additionally, the protein kinase cascades can be activated by E2 bound to mER within the cell membrane. This activation stimulates the protein tyrosine kinase/mitogen-activated protein kinase (Boonyaratanakornkit and Edwards, 2007) and occurs through interactions with the metabotropic glutamate receptors (Tonn Eisinger et al., 2018).

## 2 Effects of estradiol on neurotransmitter systems

E2 has demonstrated an impact on crucial systems involved in mood regulation and memory systems, including the serotonergic, dopaminergic, and glutamatergic systems in both sexes, suggesting that targeting the E2-ER system could present novel therapeutic approaches for treating impairments associated with dysfunction of these systems. It is crucial to acknowledge that the effects observed in different hormonal contexts or with hormone replacement therapy may not solely be attributed to estradiol or estrogens alone. Other steroid hormones could contribute to or modify these effects, highlighting the complexity of hormonal interactions and their implications for physiological processes or therapeutic interventions. However, the effects of other hormones are beyond the scope of this review. Below we discuss the findings supporting an interaction between E2 and these systems.

### 2.1 Serotonin

Previous findings indicate a potential role of E2 and ERs in the serotonergic system, which is implicated in mood regulation and consequently the development of mood disorders such as depression and anxiety (Baldwin and Rudge, 1995; Hernández-Hernández et al., 2019). While ERα predominantly expresses in regions dedicated to reproductive function in the body and brain, ERβ is found in diverse brain areas, notably the hippocampus and amygdala, both relevant to learning and memory and emotional regulation (Mitra et al., 2003; Imwalle et al., 2005). Additional studies reveal ERβ expression in the dorsal raphe nucleus (DRN) of male and female mice (Alves et al., 1998). The DRN, housing a third of the brain’s serotonergic neurons, assumes a pivotal role in the serotonergic system (Jacobs, 2010). It is responsible for serotonergic projections (5-HT) to various brain areas, including the hippocampus and amygdala.

Tryptophan hydroxylase (TPH) serves as a rate-limiting enzyme in serotonin synthesis (Kuhn and Hasegawa, 2020). TPH exists in two isoforms: TPH-1, predominantly expressed in the periphery, and TPH-2, found in neuronal cells (Walther and Bader, 2003). An investigation used the serotonergic cell line, B14, derived from embryonic rat medullary raphe cells which endogenously expresses ERβ but not ERα, and transfected with human TPH2-luc to investigate the role of E2 and ERβ in TPH2 activity (Hiroi and Handa, 2013). Subsequent treatment of TPH2-luc cells with E2 or R-DPN, the more active isoform of diarylpropionitrile (an ERβ agonist), resulted in increased TPH2-luc activity in B14 cells, suggesting ERβ utilizes genomic mechanisms to induce TPH2 transcriptional activity. B14 cells also exhibited an ERE half-site between nucleotides −792 and − 787, the DNA section for TPH-2 transcription, which was hindered by an ERβ antagonist (Hiroi and Handa, 2013). This implies that ERβ must be active for TPH-2 transcription to occur. In line with this, it was shown that female ERβ knockout mice showed significantly lower serotonin levels in several brain regions, including the hippocampus and nucleus accumbens (NAc), compared to wild-type mice (Imwalle et al., 2005). Male ERβ knockout mice also experienced a statistically significant decrease in TPH mRNA expression in the DRN compared to both wild-type and ERα knockout mice (Nomura et al., 2005) whereas administration of an ERβ agonist and E2 increased expression of *tph2* in the DRN (Donner and Handa, 2009). Likewise, Suzuki et al. demonstrated that ERβ but not ERα is strongly expressed in the DRN and there was a significant decrease in the number of TPH-positive normal-looking neurons and a significant increase in TPH-positive spindle-shaped cells in ovariectomized (OVX), WT, and ERβ knockout mice (Suzuki et al., 2013). Treatment with the selective ERβ agonist, LY3201, for 1–3 weeks prevented these neuronal changes (Suzuki et al., 2013). These findings suggest that ERβ, rather than ERα, is involved in TPH transcriptional activity and, consequently, serotonin synthesis. It has also been proposed that acute tryptophan depletion has led to relapse in patients recovered from depression and induced depressive symptoms in healthy subjects (Albert et al., 2011). This is noteworthy because estradiol regulates the expression of TPH-2, which is then converted to 5-HT by the tryptophan hydroxylase enzyme and further decarboxylated to produce serotonin.

Furthermore, E2 may impact the serotonergic system through interactions with the serotonin reuptake transporter (SERT). SERT facilitates the reuptake of excess extracellular 5-HT from the synaptic cleft to the presynaptic terminal (Kanova and Kohout, 2021). Selective serotonin reuptake inhibitors (SSRIs), which block SERT, increase synaptic serotonin concentration. SSRIs are the first-line treatment for major depressive disorder, and their effectiveness is attributed to the elevation of 5-HT neurotransmission. This results in reduced 5-HT cell firing, activation of 5-HT_1A_ receptors, and terminal 5-HT release (Imwalle et al., 2005). Prior research indicates that both depressed males and females show decreased SERT in the diencephalon, but females experience a more pronounced decrease, possibly explaining the increased antidepressant effect in females from SSRIs compared to males (Staley et al., 2006). An aggregation of published studies reveals varying effects of E2 on SERT. While a majority of studies indicate that E2 upregulates SERT expression across numerous brain regions, there are some disparities in the findings (Hudon Thibeault et al., 2019). Specifically, in female OVX mice, SERT binding density significantly decreases in the hippocampus compared to wild-type mice (Bertrand et al., 2005). In agreement, OVX macaques reveal reduced SERT expression intensity and the number of 5-HT-positive cells in the rostral dorsal raphe compared to intact animals (Bethea et al., 2011). OVX rats treated with estradiol benzoate, an E2-based estrogen medication, exhibit significantly increased SERT mRNA expression in the DRN and heightened SERT-binding site density in various brain areas, including the ventromedial hypothalamic nucleus (VMN; McQueen et al., 1997). Altogether these findings suggest a correlation between E2 and SERT function and expression, consequently suggesting that E2 may be one of the contributing mechanisms for the antidepressant effect of SSRIs.

An extensive investigation was performed regarding estrogen’s impact on the brain serotonergic system, suggesting that gene expression for serotonin transporter and monoamine oxidase A are decreased and, tryptophan hydroxylase and serotonin receptor 2A (5-HT_2A_ receptor) are increased (Hildebrandt et al., 2010; Hudon Thibeault et al., 2019). The activation of 5-HT_1A_ receptors reduces the firing activity of 5-HT neurons (Riad et al., 2001). E2 suppresses 5-HT_1A_ receptor signaling by uncoupling 5-HT_1A_ receptors from their associated G protein (Raap et al., 2002; Mize et al., 2003), indicating a potential increase in the activity of 5-HT neurons. Similarly, Adeosun et al. demonstrated the impact of the non-genomic mechanism of E2 on the 5-HT_1A_ protein. Using SH SY5Y neuroblastoma cells, known for expressing TPH-2 and 5-HT_1A_, it was shown that E2 downregulated the presence of 5-HT_1A_ and upon withdrawing E2, 5-HT_1A_ levels returned to normal. 5-HT_1A_ lacks ERE in the promoter region suggesting mERs or G-protein coupled receptor Gα_q_ may be responsible for the effect E2 has on regulation of the protein (Adeosun et al., 2012). Moreover, it was shown that the desensitization of 5-HT_1A_ receptors that is necessary for SSRI therapeutic efficacy in the paraventricular nucleus of the hypothalamus in rats, depends on GPER (McAllister et al., 2012). Notably, in sl6a4−/− mice with reduced 5-HT concentration in the brain, the density of 5-HT_1A_ receptors decreases more significantly in females than in males (Li et al., 2000), suggesting a potential promotion of the effects of 5-HT on 5-HT_1A_ receptor expression by female hormones. GPER and 5-HT_1A_ receptors were found to be co-expressed in the hypothalamus in rats, further supporting this concept (Lu et al., 2009; Xu et al., 2009; Akama et al., 2013). Nonetheless, E2 treatment, in cells transfected with 5-HT_1A_ receptors and ERα promoters, increased 5-HT_1A_ receptors levels through ERα and NF-κB activation suggesting that NF-κB collaborates with ERα to recruit cofactors that collectively enhance activity of 5-HT_1A_ receptor gene promoter (Wissink et al., 2001).

Post-synaptic 5-HT_2A_ receptors serve as excitatory neuron receptors. E2 increases 5-HT_2A_ receptor levels in the brains of post-menopausal females (Kugaya et al., 2003; Moses-Kolko et al., 2003). *Rapkin et*. Activation of 5-HT_2A_ receptors induces transient desensitization of 5-HT_1A_ receptor signaling (Zhang et al., 2001). It is theorized that after 5-HT_2A_ receptor activation, 5-HT1_A_ receptors become unable to reduce 5-HT production, resulting in an increase in 5-HT concentrations (Rybaczyk et al., 2005). Additionally, an increase in the firing rate of DRN serotonergic neurons was observed in OVX rats treated with E2 (Robichaud and Debonnel, 2005). Rybaczyk et al. suggest that the balance between 5-HT_1A_ and 5-HT_2A_ receptor signaling contributes to different pathophysiological changes depending on which system is affected (Rybaczyk et al., 2005).

Moreover, it was suggested that E2 affects the brain’s serotonergic system through its actions on ERβ (Gupta et al., 2011). In ERβ knockout mice, 5-HT levels are lower in various brain regions compared with controls (Imwalle et al., 2005). Likewise, in the rodent dorsal raphe nucleus, tph1 levels were heightened by E2, an effect mediated by ERβ (Gundlah et al., 2005; Nomura et al., 2005). Additionally, E2 administration induced reduction of serotonin clearance through ERβ activation (Benmansour et al., 2014). This effect required activation of the MAPK/ERK1/2 signaling pathways and implicated interactions both with TrkB and IGF-1R (Benmansour et al., 2014). Using rat derived serotonergic neuronal cell line that expresses SERT and ERβ but lacks ERα, it was shown that E2 application resulted in a reduction of SERT activity (Koldzic-Zivanovic et al., 2004).

Several studies have indicated a correlation between estrogen and serotonin levels during the menstrual cycle. During the follicular phase, when there is an abundancy of estrogen, there is an increase in serotonin levels. The opposite has been seen during the luteal phase, where estrogen levels are low and progesterone levels are high, there is a decrease in serotonin levels (Rapkin et al., 1987; Sacher et al., 2023). Moreover, it was suggested that in the presence of both E2 and progesterone (representing pre-menopausal stage) 5-HT_2A_ receptor density increases, while only E2 (representing post-menopausal stage) increases 5-HT_1A_ receptor signaling (Rybaczyk et al., 2005). Steroid hormones can also influence levels of the serotonin precursor, tryptophan. For example, E2 administration or anti-androgen treatment in male-to-female transsexuals, resulted in decreased tryptophan levels, whereas in the opposite situation testosterone administration enhanced tryptophan levels (Giltay et al., 2008).

### 2.2 Dopamine

Like serotonin, dopamine (DA) is a neurotransmitter implicated in reward processing and mood regulation (Diehl and Gershon, 1992; Belujon and Grace, 2017). Tyrosine hydroxylase (TH), similar to TPH in serotonin synthesis, is a rate-limiting enzyme in the synthesis of catecholamines, including DA (Paravati et al., 2023).

Both ERα and ERβ have been implicated in the transcription of TH. A study examined PC12 cells, a type of catecholamine cells that synthesize, store and release dopamine, transfected with the reporter construct p5’TH (−773/+27)/Luc and expression vectors for mouse ERα and ERβ, investigating ERs’ influence on TH transcription. The reporter constructs with luciferase illuminated ER activity during TH transcription. PC12 cells treated with varying E2 concentrations (ranging from 0.01 nm E2 to 10 nm E2) showed increased reporter activity in ERα at higher concentrations, while ERβ exhibited decreased reporter activity by 25–50% at any E2 concentration (Maharjan et al., 2005). This suggests that both ERα and ERβ affect TH transcription rates, potentially working in tandem to maintain TH concentration at homeostatic levels. The study also observed that genetic removal of the putative half ERE did not alter E2’s effect on TH transcription (Maharjan et al., 2005), indicating the possibility that E2’s transcriptional regulation of TH might occur through nongenomic mechanisms.

Moreover, while estradiol exerts notable modulatory effects on DA systems in females, there is no evidence supporting its ability to enhance striatal DA release in males (Becker, 1990, 2005; Yoest et al., 2014, 2018). Despite recent advancements, the precise mechanism through which E2 influences DA systems remains not well understood. E2 exhibits both acute and chronic effects on functional activity, impacting DA release, reuptake, and downstream targets of DA receptor activation.

Exogenous E2 administration demonstrates a biphasic effect on dopamine D2 receptor (D2) expression. Apart from its acute downregulation of D2 binding in OVX females, chronic E2 has been observed to enhance D2 binding in the striatum and NAc core, likely mediated by its effect on ERβ (Bazzett and Becker, 1994; Le Saux et al., 2006). In contrast with these findings, no change was observed in DA D2 receptor availability in human females in the whole striatum and its subregions during the high-estrogen vs. low-estrogen phases of the menstrual cycle (Petersen et al., 2021).

In female OVX rats, the acute administration of E2 was shown to elevate levels of the dopamine metabolite, dihydroxyphenylacetic acid (DOPAC), in the prefrontal cortex (PFC; Inagaki et al., 2010). In agreement with this, Pasqualini et al. demonstrated that acute exposure to E2 rapidly enhances DOPAC concentrations (Pasqualini et al., 1995). However, it is important to note that this increase was associated with alterations in DA synthesis and not with escalation in DA release (Pasqualini et al., 1995). It was also revealed that the administration of physiological doses of E2 not only boosted the synthesis of DA and DOPAC but did so without causing a change in their overall concentrations within the striatum. The mechanism behind this effect was suggested to involve a reduction in the inhibitory impact of DA on TH, possibly through enzyme phosphorylation (Pasqualini et al., 1995).

Long-term treatment with E2 or the selective ERβ agonist diarylpropionitrile (DPN) prevented the decrease in dopamine transporter (DAT) expression in the medial striatum caused by OVX in mice, with no significant impact on mRNA expression. However, these changes might be outcomes of an enhancement in DA release and not a direct E2 effect on receptor expression (Morissette et al., 2008). DAT activity is known to be regulated by kinases including PI3K, MAPKs, PKA, and PKCs. PC12 cells treated with kinase inhibitors for PI3K, MAPK, PKA, and PKC and E2 at a dose of 10^−9^ M showed significant inhibition of E2-mediated dopamine influx in all groups except the PI3K and PKA inhibited cells (Alyea et al., 2008; Alyea and Watson, 2009). This suggests the importance of PI3K and PKC activation in E2-mediated dopamine efflux and the use of non-genomic mechanisms to achieve E2-mediated dopamine influx. Acute E2 treatment has been demonstrated to increase the amphetamine-induce DA response in the striatum of females, both *in vitro* and *in vivo*, but not in males (Becker, 1990; Castner et al., 1993). Additionally, acute E2 treatment enhances K + -stimulated DA release in both the striatum and NAc (Becker, 1990; Thompson and Moss, 1994). Moreover, it was shown that the administration of E2 to the striatum, but not the medial PFC or substantia nigra, increased DA release induced by amphetamine administration and electrical stimulation, in OVX female rats (Shams et al., 2016, 2018). Interestingly, elevated baseline DA levels were observed during estrus, coinciding with a decline in estrogen levels, and reduced basal dopamine levels during proestrus, when estrogen levels peak (Dazzi et al., 2007). Notably, rats in proestrus display heightened ethanol-induced dopamine release in the PFC, suggesting a dual action of estrogens: decreasing basal DA levels while enhancing DA release in this brain region (Dazzi et al., 2007). Moreover, increased in ventral tegmental area DA neuron excitability was revealed during estrus compared with proestrus and metestrus (Shanley et al., 2023). Remarkably, the impact of E2 on DA release in response to cocaine was attenuated following lesions of the medial preotic area in rats, an effect that was reversed by E2 microinjections in the medial preotic area (Tobiansky et al., 2016). In line with these findings, male ERα knockout mice showed decreased in the hyperlocomotion induced by amphetamine, compared wildtype mice, suggesting a possible interaction between ERα and dopaminergic signaling (Georgiou et al., 2019). The likely interplay between ERα and the dopaminergic system is further corroborated by observations indicating that male mice lacking ERα exhibit decreased levels of TH mRNA and protein in the midbrain (Küppers et al., 2008), which may also explain the finding that male ERα knockout mice exhibit decreased response to amphetamine-induced hyperlocomotion.

Moreover, accumulating evidence suggests a clear role of mERs in DA neurotransmission. The rapid augmentation of both tonic and phasic DA release by E2 has been documented (Shams et al., 2016, 2018), alongside its ability to decrease calcium currents via mER, particularly in the dorsal striatum. Moreover, while systemic E2 injections administered 48 h prior to testing lead to decreased phasic DA release in the NAc, direct infusion of E2 into the NAc enhances phasic DA release within 15 min (Thompson and Moss, 1994), suggesting opposing genomic and nongenomic effects of E2 on DA release. Also, rapid effects of E2 on NAc DA was observed, with systemic administration of E2 resulting in increased cocaine-evoked DA release within 30 min (Yoest et al., 2018). Additionally, E2 promptly boosted DA metabolism in the NAc, evidenced by elevated levels of DOPAC and homovanillic acid within 30 min of administration (Di Paolo et al., 1985).

Additionally, light and electron microscopy in the dorsal striatum revealed the presence of ERα, ERβ at nonnuclear sites, particularly in GABAergic and, to a lesser extent, cholinergic and catecholaminergic neurons (Almey et al., 2012, 2016, 2022) and previous studies utilizing light microscopy and *in situ* hybridization have reported minimal ERα and ERβ immunolabeling in the adult rodent NAc, predominantly at nuclear sites (Shughrue et al., 1998; Mitra et al., 2003; Lorsch et al., 2018; Krentzel et al., 2021; Quigley and Becker, 2021). GPER1 is also detected at low levels in the perikaryal cytoplasm of the adult rodent NAc, likely localized to cellular organelles and the plasma membrane (Hazell et al., 2009), as previously observed (Funakoshi et al., 2006; Otto et al., 2008). Nuclear ERα, ERβ, and GPER1 binding by estrogens could underlie the genomic effects in the NAc, while GPER1 might contribute to some nongenomic effects. However, the rapid nongenomic effects of estrogens in the NAc may also involve membrane associated ERα or ERβ. Further investigation, possibly through ultrastructural analysis, is warranted to determine the presence and localization of mERα, mERβ, and GPER1 in the NAc and their distribution among various cell types.

E2 may also exert downstream effects on the mesolimbic DA system. Specifically, pre-treatment with E2 in cells decreases D2 receptor inhibition and increases D1 receptor activation of adenylate cyclase activity (Maus et al., 1989). Furthermore, E2 treatment reduces the expression of Regulator of G protein Signaling 9–2, which is associated with D2 receptor signaling (Silverman and Koenig, 2007).

Findings by Mermelstein et al. suggest that E2 might influence DA release through GABA medium spiny neurons (Mermelstein et al., 1996). Tissue application of E2 acutely decreases Ca^2+^ currents in GABAergic striatal neurons in females but not males. This effect is also observed with E2 conjugated with and is not reversed by prevention of GTP inactivation, suggesting that E2 acts on a G-protein coupled receptor on the cell membrane (Mermelstein et al., 1996). Moreover, bovine serum albumin conjugated E2 exert the same effects as E2 in enhancing amphetamine-induced striatal dopamine release, supporting the presence of striatal mERs since conjugated E2 cannot cross the cell membrane (Xiao and Becker, 1998).

While investigations into the impact of E2 on DA release in males revealed limited effects, there is evidence of E2 influencing DA receptor sensitivity. In a manner akin to OVX females, intact male rats administered with a single dose of E2 valerate, which undergoes slow metabolism over 6 days in rats, exhibited heightened sensitivity to the apomorphine-induced stereotypical effects 6 days later. Additionally, they displayed an extended duration of rotating behavior induced by amphetamine (Hruska and Silbergeld, 1980a). Unlike females, males did not exhibit an acute decrease in DA receptor sensitivity or alterations in DA receptor binding affinity (Hruska et al., 1980; Hruska and Silbergeld, 1980b). Instead, DA receptor sensitivity changes in males following E2 valerate administration attributed to modifications in the expression of DA receptors within the striatum. The increase in DA receptor expression (Bmax) was observed 3 days after E2 administration (Hruska and Silbergeld, 1980a,b). Intriguingly, these alterations in DA receptor sensitivity were nullified after hypophysectomy, suggesting that estradiol indirectly influenced DA receptor expression in males, potentially through the mediation of prolactin (Hruska et al., 1980).

The expression of ERα and ERβ in dopamine neurons has been identified in the basal ganglia (Shughrue et al., 1998), an area of the brain rich in dopaminergic neurons and responsible for decision-making, motivation, reward, and addiction. Administration of E2 to 2-week-old OVX rats significantly increased the density of dopamine receptors in the dorsal lateral striatum; however, a non-significant increase in DA receptors was observed in the dorsomedial caudate-putamen (Falardeau and Di Paolo, 1987).

Like estrogens, progesterone and its receptors are present throughout the dopaminergic system and has been shown to affect several brain regions including the amygdala and striatum. Progesterone has been implicated in the activation of the amygdala-hippocampal complex in regard to reward anticipation in humans. During the luteal phase of the menstrual cycle, progesterone is correlated with the activation of the right amygdala-hippocampal complex with E2 playing a limited role, while during the follicular phase of the menstrual cycle E2 is correlated with the activation of this complex (Dreher et al., 2007). This change in activating hormones through the menstrual cycle may be due to an increase in progesterone during the luteal phase and a decrease during the follicular phase and an inverse in E2 levels during the same phases. Periods of low progesterone levels, such as proestrus, have been shown to increase vulnerability to cocaine self-administration in female OVX rats. Treatment with progesterone reduced the amount of cocaine voluntarily administered to the rats suggesting that in reward-seeking behaviors, such as cocaine usage and addiction, lower levels of progesterone, which can be seen during post-menopause, are correlated with an increase in reward-seeking behaviors and administration of progesterone. E2, simulating the pre-menopausal stage, has shown a negative correlation with self-administered cocaine consumption suggesting an effect on the dopaminergic reward system (Harp et al., 2020).

These studies implicate E2 as an impactful hormone in the dopaminergic system, specifically in brain regions associated with reward and mood regulation, such as the striatum, NAc, and HPA axis. Further research will contribute to a better understanding of how E2 and ER play a role in DA function.as it pertains to regular reward functioning and reward-associated disorders.

### 2.3 Glutamate

Glutamate, the primary excitatory neurotransmitter in the brain with a seminal role in neurotransmission and the development of psychiatric disorders. Its complex interplay with estrogen has become a focal point in understanding the molecular dynamics that underlie mood regulation and mental health. Interestingly, evidence supports the neuroprotective role of estrogen against diseases and injuries affecting the nervous system. For instance, Chen et al. (1998) demonstrated the protective properties of E2 on CA1 hippocampal cells following ischemia in gerbils. Moreover, estrogen was recognized for its unique role in the nervous system, with contributing to synaptic function (Wong and Moss, 1992; Woolley and McEwen, 1992; Warren et al., 1995; Murphy and Segal, 1996; Córdoba Montoya and Carrer, 1997; Murphy et al., 1998; Pozzo-Miller et al., 1999). Such synaptic function is deemed essential for emotional behavior, learning, and memory (Brown et al., 1988; Tsien and Malinow, 1991; Tsumoto, 1992; Bliss and Collingridge, 1993; Izquierdo, 1994; Maren, 1999; Blair et al., 2001; Ito, 2001). However, the exploration of estrogen-mediated synaptic plasticity has been somewhat limited.

Foy et al. (1999) reported that E2 treatment rapidly enhanced NMDA glutamate receptor-mediated excitatory postsynaptic potential and hippocampal long-term potentiation. In the hippocampal CA1 region of OVX rats, chronic E2 treatment not only increased dendritic spine density (Woolley and McEwen, 1994) but also augmented the number of NMDA receptor binding sites (Weiland, 1992; Gazzaley et al., 1996). Moreover, estrogens enhance kainate-induced currents in hippocampal neurons from both wild-type (Gu and Moss, 1998) and estrogen-receptor ERα knockout (Gu et al., 1999) mice. Additionally, they prompt rapid formation of spine synapses in the CA1 hippocampus of OVX rats (MacLusky et al., 2005). Moreover, the acute application of estrogens to hippocampal slices results in increased transmission of NMDA and AMPA receptors (MacLusky et al., 2005), induces both long-term potentiation (LTP) and long-term depression (LTD; Good et al., 1999), and modulates neuronal excitability in the rat medial amygdala (Nabekura et al., 1986) and hippocampus (Nabekura et al., 1986). These findings suggest the potential involvement of estrogen in neuronal plasticity through the potentiation of postsynaptic function.

While the significance of postsynaptic function is acknowledged, the importance of the presynaptic system in neuronal plasticity is also crucial. Zakharenko et al. (2001) demonstrated that short-term and long-term synaptic plasticity at the CA3-CA1 synapse in acute hippocampal slices may, in part, depend on enhanced transmitter release from presynaptic neurons. Consequently, the regulation of presynaptic function could be critical for changes in synaptic transmission. However, the contribution of estrogen to presynaptic function remains incompletely understood, and the signaling mechanisms governing estrogenic regulation of synaptic transmission are yet to be elucidated.

Moreover, it has been shown that estradiol enhances depolarization-induced glutamate release by up-regulating the exocytotic machinery (Yokomaku et al., 2003). Conversely, it has no effect on the release of GABA induced by high potassium (HK+)-evoked depolarization (Yokomaku et al., 2003). This enhanced release by estradiol is contingent on the activation of phosphatidylinositol 3-kinase (PI 3-kinase) and MAPK (Yokomaku et al., 2003). Collectively, these findings suggest that estrogen augments excitatory synaptic transmission by increasing the release of the neurotransmitter glutamate. In support of this, in a period of low E2, such as perimenopause or menopause, concentrations of glutamate within the medial prefrontal cortex also decrease. The influence concentrations of E2 have on glutamate levels may explain the increase in depression that comes with age (Yap et al., 2021). Additionally, it was demonstrated that a high physiological dose of estradiol (200 pM) significantly increased firing rate and miniature postsynaptic current frequency during proestrus in hypothalamic GnRH neurons, with ERβ inhibition blocking these effects indicating that it is key player in mediating estradiol’s facilitatory glutamate neurotransmission effect (Farkas et al., 2018). Similarly, it was shown that estradiol increases kisspeptin 1 neuronal excitability and glutamate neurotransmission in the hypothalamus in females (Qiu et al., 2018). Moreover, a decrease in glutamatergic neurons and an increase in GABAergic neurons was observed in the preoptic area in OVX rats, with a concomitant decrease in both ERα and β expression. Estrogen administration reversed these changes demonstrating that the decreased in estradiol was responsible for the decrease in glutamatergic neurons (Sun et al., 2022). Also, in male orchiectomized (ORX) mice, a decrease in neuronal activity was observed in response to social reward stimuli in the ERβ-expressing glutamatergic projection from the basolateral amygdala to NAc following acute stress (Georgiou et al., 2022). These observations imply that estradiol modulates glutamatergic activity via ERβ, influencing the subsequent responsiveness to rewarding stimuli, and may represent one of the fundamental mechanisms contributing to depressive phenotypes.

In addition to the effects of estrogens on ionotropic glutamate receptors and synaptic plasticity, an interaction between metabotropic glutamate receptors (mGluRs) and membrane-localized estrogen/estrogen receptors were observed. Boulware et al. used CA3-CA1 hippocampal pyramidal neurons from male and female rat pups to better understand how E2 is affecting mGluRs. Within 5 min of introducing E2 into the pyramidal neurons, there was a significant increase in cAMP, response element-binding protein (CREB; Boulware et al., 2005), a transcription factor that has been implicated in addiction within the NAc (Lonze and Ginty, 2002), phosphorylation created by the MAPK/ERK. CREB phosphorylation can be activated by mGluR1a through ERα activation, while mGluR2 can limit CREB phosphorylation through both ERα and ERβ suggesting non-genomic mechanisms of ER due to phosphorylation by kinase pathways (Boulware et al., 2005). This shows that both E2 and ERs initiate mGluR signaling of CREB phosphorylation and in turn leads to transcription of genes. Also, several reports support that mGluRs are functionally coupled to the classical estrogen receptors, ERα and ERβ, through association with caveolin proteins, a group of scaffolding proteins guiding receptors to the membrane, and consequently, regulate the E2 membrane effects (Mermelstein, 2009; Martinez et al., 2014; Tonn Eisinger et al., 2018). The mechanism of action of mER involves glutamate-independent transactivation of mGluRs, resulting in a variety of signaling outcomes (Johnson et al., 2022). Specifically, caveolin 1 is responsible for the interactions of mERα with mGluR1 or mGluR5, and this transactivation leads to the activation of MAPK/cAMP, which results in Ca^2+^ release. The interactions of caveolin 3 with mERα or mERβ are associated with mGluR2 and mGluR3 when cAMP and Ca^2+^ flux is reduced (Mermelstein, 2009; Martinez et al., 2014; Barabás et al., 2018; Tonn Eisinger et al., 2018). Moreover. the activation of mGluR1a through ERβ by E2 has been identified in male quails to regulate sexual behavior, and both males and females have been shown to display mER-mGluR signaling in the cerebellum (Cornil et al., 2012; Seredynski et al., 2015; Hedges et al., 2018). Additionally, E2 binding to ERα activates mGluR5, leading to the activation of the Gq subunit and increased CREB phosphorylation. Simultaneously, E2 binding to both ERα and ERβ-associated mGluR3 hinders CREB phosphorylation due to the Gi/o subunit activation (Mermelstein, 2009). The functional interaction of these receptors seems to be driven by caveolin proteins, with caveolin 1 for mGluR5 and caveolin 3 for mGluR3 (Boulware and Mermelstein, 2009; Mermelstein, 2009). Additionally, in diestrus where E2 levels are low, mERα-mediated activation of mGluR1 suppressed spinal endomorphin 2 antinociception. However, in proestrus, which E2 levels are high, spinal endomorphin 2 antinociception was facilitated independently of mERα, namely, through glutamate-activated mGluR1 (Liu et al., 2019). Altogether, these findings underscore the significance of the interaction between mER and glutamate receptors in the regulation of neuronal activity and emphasize the need for further investigation to delineate their roles in physiological functions and in disease states.

Similar to serotonin and dopamine, glutamate is affected by the presence of progesterone. When working in conjunction with E2, progesterone increases the expression of transporters GLT-1 and EAAT3 which are responsible for glutamate uptake (Nematipour et al., 2020). When observed separate from E2, progesterone can activate the neuroprotective mitogen-activated protein kinase and phosphoinositide-3 kinase pathways in order to decrease glutamate-induced toxicity (Goyette et al., 2023). Progesterone also plays a role in glutamate release. When reacting with 5α-reductase, progesterone produces 5α-dihydroprogesterone which can be converted into allopregnanolone through 3-hydroxysteroid oxidoreductase (Reddy, 2010). During the luteal phase of the menstrual cycle and menopause, the abundance of progesterone is higher than that of estrogens which in turn can increase the amount of circulating allopregnanolone (Giacometti et al., 2022). Allopregnanolone plays a role in glutamate release and potentially glutamate uptake in the peripheral nervous system, but more research is needed to better understand the role of progesterone in glutamate release (Perego et al., 2012; Goyette et al., 2023). One study presents a correlation between plasma estrogen and progesterone and glutamate levels in the blood in humans. They found that when blood samples were taken while estrogen and progesterone levels were low, as seen during menstruation, glutamate levels were high. Similarly, their results indicate that when estrogen and progesterone levels are high, blood glutamate levels drop to nearly half the value seen at the beginning of the menstrual cycle (Giacometti et al., 2022). Another study found that an intravenous treatment of E2 prior to local application of glutamate lead to a significant reduction in the size of the lesion caused by glutamate in rats (Jincao et al., 2001). While these mechanisms remain unclear, overall, the results suggest that E2 may play a role in glutamate regulation in the plasma.

In summary, the interplay between E2 and glutamate reveals a nuanced relationship in neuronal dynamics, encompassing neuroprotection, synaptic plasticity, and excitatory neurotransmission (for summary of main effects see Table 1). Despite substantial strides in understanding postsynaptic effects, the impact on presynaptic mechanisms and the underlying signaling pathways remain areas warranting further exploration.

**Table 1:** Summary of the main findings on the role of E2 on serotonin, glutamate, and dopamine systems.

**Study Type**	**Neurotransmitter System**	**E2 Effects**	**Relevant Brain Regions**	**References**
Human Studies	Serotonin	Increases 5-HT_2A_R binding	PFC	Kugaya et al. (2003) and Moses-Kolko et al. (2003)
Rodent Studies	Serotonin	Effects to Up-regulates SERT mRNA expression and SERT binding density	DRN, BLA, ventral nucleus of the thalamus, and ventromedial hypothalamic nucleus	McQueen et al. (1997) and Hudon Thibeault et al., (2019)
		Modulates 5-HT1AR signaling	Hippocampus, Hypothalamus, PFC	Mize et al. (2003) and Raap et al. (2002)
	Dopamine	Biphasic effects on D2 receptor binding	Striatum	Bazzett and Becker (1994) and Le Saux et al. (2006)
		Enhances DA release in females, but not in males	Striatum	Becker (1990), Shams et al. (2018) and Castner et al. (1993)
		Increases D2 receptor sensitivity in males	Striatum	Hruska and Silbergeld et al. (1980)
		Reduces the expression of RGS9-2, a GTPase-activating protein associated with D2 receptor signaling.	NAc	Silverman and Koenig (2007)
	Glutamate	Increases dendritic spine density and NMDA receptor binding sites	CA1 hippocampus	Woolley and McEwen (1994) and Gazzaley et al. (1996)
		Modulates glutamatergic activity.	BLA to NAc pathway.	Georgiou et al. (2022)
		Increases neuronal activity and glutamate neurotransmission	Hypothalamic GnRH and kisspeptin neurons	Farkas et al. (2018) and Qiu et al. (2018)
		Farkas et al. (2018), Qiu et al. (2018)	Striatum	Martinez et al., (2014) and Grove-Strawser et al., (2010)
Rodent Brain Slices	Glutamate	Enhances NMDA receptor-mediated excitatory postsynaptic potential and hippocampal long-term potentiation	Transverse hippocampus slices	Foy et al. (1999)
		Enhances depolarization-induced glutamate release	Hippocampus	Yokomaku et al. (2003)
Cell Cultures	Serotonin	Increases TPH-2 expression and activity	DRN, hippocampus	Hiroi and Handa (2013)
		Modulates 5-HT1AR signaling	Hippocampus, DRN	Wissink et al. (2001)
	Dopamine	Both ERα and ERβ influence TH transcription rates	PC12 cells	Maharjan et al. (2005)
		Decreases D2 receptor inhibition of adenylate cyclase activity and enhance	Decreases D2 receptor inhibition of adenylate cyclase activity and enhance	Maus et al. (1989)
	Glutamate	Coordinates membrane effects through mGluR5 and mGluR3, associated with CAV1 and CAV3 proteins, respectively.	Striatum	Boulware and Mermelstein (2009)
		Modulates mGluR signaling and CREB phosphorylation	CA3-CA1 hippocampus	Boulware et al. (2005)

Abbreviations: 5-HT_1A_R: Serotonin receptor 1A; 5-HT2_A_R: Serotonin receptor 2A; BLA: Basolateral amygdala; CA1: Hippocampal area cornu ammonis area 1; CA3: Hippocampal area cornu ammonis area 3; CAV: Caveolin; CREB: cAMP-response element binding protein; DA: dopamine; DRN: Dorsal raphe nucleus; D2: Dopamine D2 receptor; ERα: Estrogen receptor alpha; ERβ: Estrogen receptor beta; GTPase: nucleotide guanosine triphosphate hydrolase enzyme; mGluR: metabotropic glutamate receptor; NAc: Nucleus accumbens; NMDA: N-methyl-D-aspartate; PFC: Prefrontal cortex; RGS9-2: Regulator of G-protein signaling 9; SERT: Serotonin transporter; TH: Tyrosine hydroxylase; TPH-2: Tryptophan hydroxylase 2; VMN: Ventromedial hypothalamus.

## 3 Discussion

Estradiol is a steroid hormone that influences the serotonergic, dopaminergic, and glutamatergic systems. E2 exerts its effects through classical mechanisms by binding to nuclear estrogen receptors α, and β (Björnström and Sjöberg, 2005; Le Dily and Beato, 2018), or through nonclassical mechanisms through binding to membrane bound estrogen receptors α, β, and GPER (DeLeon et al., 2020).

One of the most investigated neurotransmitter systems impacted by E2 is the serotonergic system. E2Estradiol has been implicated in the synthesis of serotonin through its action on the classical ERβ. Specifically, it increases transcription of TPH2, the type of TPH found in neuronal cells in both males and females (Donner and Handa, 2009; Hiroi and Handa, 2013). The influence of E2 and ERβ on the transcription of neuronal TPH influences the amount of serotonin that can be synthesized, as TPH is the rate limiting factor in serotonin synthesis. E2 has also shown to affect the serotonin reuptake transporter which assists in reuptake of serotonin into the presyntaptic terminal (Kanova and Kohout, 2021). While the effects of E2 on SERT remain inconclusive there have been several reported cases suggesting a correlation between E2 and SERT expression and function in OVX animals suggesting E2 plays a role in the reuptake of serotonin in the presynaptic terminal. Several studies suggest that under OVX conditions SERT binding density, expression intensity, and 5-HT-positive cells were significantly decreased (Bertrand et al., 2005; Bethea et al., 2011). Other studies found that in OVX rats treated with E2-based estrogen medications, estradiol benzoate, increased SERT m RNA expression and SERT binding density (McQueen et al., 1997). Altogether, these findings suggest a correlation between E2 and SERT expression and function, at least in females. There is an underrepresentation of males and other gender identities in the data indicating the importance of increasing our knowledge on E2’s effect of SERT other populations as well. Additionally, E2 has been shown to affect serotonin receptors 5-HT_1A_ and 5-HT_2A_, both of which have been implicated in neuropsychiatric disorders due to their ability to be activated by 5HT. Estradiol suppresses the 5-HT_1A_ receptor signaling resulting in an increase in the firing activity of the 5-HT neurons (Riad et al., 2001; Raap et al., 2002; Mize et al., 2003) suggesting a negative correlation between E2 and 5-HT_1A_ activity. Interestingly ERα has been implicated in recruiting cofactors to enhance 5-HT_1A_ gene promoter activity (Wissink et al., 2001). 5-HT_2A_ are excitatory neuron receptors that when activated desensitize 5-HT1_A_ consequently increasing serotonin concentration (Zhang et al., 2001; Kugaya et al., 2003; Moses-Kolko et al., 2003; Rybaczyk et al., 2005). While there have been many studies examining the relationship between E2 and the serotonergic system, there are still areas that need further investigation. Attention needs to be especially paid to other populations in addition to females as there is limited information pertaining to the effects of E2 on the serotonergic system in males. A better understanding of the roles of E2 and ERs in the serotonergic system will help to gain knowledge on neuropsychiatric disorders caused by impairments of this system.

When observing the effects of E2 on the dopaminergic system, it is shown that ERα and β play a role in TH transcription. Maharjan et al., reported increased activity in ERα when the cells were under high concentrations of E2 and ERβ decreased activity at any E2 concentration (Maharjan et al., 2005), suggesting both ERα and β work in tandem to ensure TH transcription rates remain at homeostatic levels. Similar to TPH, TH is the rate-limiting enzyme in dopamine synthesis indicating that E2, ERα, and ERβ influence dopamine transcription. It is also thought that E2 exerts these effects on its receptors through nonclassical mechanisms as the half-ERE site on the DNA was genetically removed in this specific study (Maharjan et al., 2005). Apart from affecting transcription within the dopaminergic system, E2 has been shown to affect D2receptor. Several studies indicate that OVX decreases D2 binding while chronic E2 increases D2 binding in the striatum and NAc core (Bazzett and Becker, 1994; Le Saux et al., 2006). Other studies observed no change in D2 availability in the striatum during both the high and low-estrogen phases of the menstrual cycle (Petersen et al., 2021). These contradictory findings leave inconclusive results for determining the effect of E2 on D2. Future studies should not only be replicated to ensure accuracy among findings of the relationship of E2 and DA, but also should include males as they are severely underrepresented in this line of research. Dopamine transporter activity is regulated by several kinases including PI3K and PKA that do not show significant inhibition of E2-mediated dopamine influx (Alyea et al., 2008; Alyea and Watson, 2009), suggesting E2 exerts its effects on PI3K and PKA through the nonclassical mechanism in to assist in the regulation of DAT influencing the rate at which dopamine can be transported. In OVX mice long-term treatment with E2 or DPN prevented the decrease in DAT expression, although it may be due to the enhancement of DA release and not directly caused by E2 (Morissette et al., 2008). Amphetamine-induced DA response increased in the striatum (Becker, 1990; Castner et al., 1993) and K + -stimulated DA release increased in both the striatum and NAc (Becker, 1990; Thompson and Moss, 1994) *in vivo*, vitro, and OVX females when treated with E2, but not in males. It will be beneficial to future research to gain a better understanding of how E2 and ERs influence dopamine function in order to explore the impact E2 has neuropsychiatric disorders linked to dopamine dysfunction.

Finally, estradiol has been implicated in several areas within the glutamatergic system. Treatment with E2 enhanced NMDA glutamate receptor-mediated excitatory postsynaptic potential, hippocampal long-term potentiation (Foy et al., 1999), increase in dendritic spine density (Woolley and McEwen, 1994), and increased the number of NMDA receptor binding sites (Weiland, 1992; Gazzaley et al., 1996). The postsynaptic effects of E2 on these actions is necessary to understand the synaptic plasticity capabilities of estradiol. The presynaptic effects need further investigation as its function with E2 is insufficiently understood. E2 enhances depolarization-induced glutamate release by upregulating the exocytotic machinery and has no effect on the high-potassium induced release of GABA, glutamate’s counterpart (Yokomaku et al., 2003). A decrease in E2 levels correlates with the decrease of glutamate concentrations within the medial PFC (Yap et al., 2021). High doses of E2 significantly increased firing rate and miniature postsynaptic current frequencies in hypothalamic GnRH neurons. ERβ inhibition blocked these increased effects indicating the importance of ERβ in facilitating glutamate’s neurotransmission effect (Farkas et al., 2018). OVX rats experienced a decrease in glutamatergic neurons in the preoptic area and a decrease in both ERα and β expression (Sun et al., 2022). ORX mice experienced a decrease in neuronal activity in response to social reward stimuli following acute stress in the ERβ-expressing glutamatergic projection from the BLA to NAc (Georgiou et al., 2022). Both of these findings suggest E2 modulates glutamatergic activity via ERβ. Metabotropic glutamate receptors and mERs have been observed in interaction with E2. ERα, in the presence of E2, has the ability to achieve transcription through activation of mGluR1a to initiate CREB while mGluR2 initiates CREB phosphorylation through both ERs (Boulware et al., 2005) to achieve transcription. CREB can then be inhibited through the binding of E2 to both ERs associated with mGluR3. mGluRs have also been reported to be coupled to classical ER through association with caveolin proteins and in turn may regulate E2’s effects (Mermelstein, 2009; Martinez et al., 2014; Tonn Eisinger et al., 2018). This evidence suggests that E2 may regulate mGluRs and consequently the reception of glutamate. Further investigation into the role E2 plays in the glutamatergic system will help to better understand the impact of E2 in brain disorders caused by the impairment of the glutamatergic system in both females and males.

In summary, the effects of E2/ERs on neurotransmitter systems is vast, complicated, and more research is necessary. While E2 is mainly thought of as a female sex hormone, the culmination of evidence implicating E2 in neurotransmitter system function and regulation is heavily evident. Further investigation into the effects of E2 on the serotonergic, dopaminergic, and glutamatergic system will not only help to solidify contradictory and inconclusive findings but help to better understand the influence of E2 in underrepresented populations.

## 4 Conclusion and future directions

In conclusion, we provide a comprehensive review of the many effects of E2 on neurotransmitter systems and more specifically we provide evidence supporting the hypothesis that E2 may enhance serotoninergic, dopaminergic and glutamatergic neurotransmission. Investigating animal, human, and cellular data has proven beneficial in understanding how, where, and why E2 exerts its effects on both the male and female brain. Further investigation into the effects estradiol on the brain, specifically in males and intersex individuals is required to address the current knowledge gabs. Moreover, the investigation on how the estrogenic regulation of this neurotransmitter systems may affect or underlie pathophysiological states needs to be further explored. Specifically, targeting the etrogenic system might be a novel approach for the treatment of mood disorders, learning and memory deficits as well as schizophrenia.

Also, it is important to state that there is a huge variety of approaches used to examine the role of the estrogen system in the brain and their behavioral outcomes which makes conclusions challenging. Standardizing methodologies including hormone manipulation, neuronal manipulations, and behavioral studies will allow for consistency among results and will result to more robust conclusions. Expanding research to include diverse populations with varying age ranges, sex and gender identities will allow for the most comprehensive analysis of future data. Additionally, more studies on the effects of genomic vs. non-genomic actions of ERs are necessary, especially pertaining to hypogonadal conditions like aging. It was shown that in female rats there is a negative correlation with mERα and mERβ trafficking and aging within the synapses of the hippocampus (Adams et al., 2002; Waters et al., 2011). The decline in mER functioning with age may partially explain why there is an increase in depression and depression-like symptoms in post-menopausal females and increase in Alzheimer’s and related dementias (Jamshed et al., 2014; Hidalgo-Lopez and Pletzer, 2017). Future research is needed to determine the role of estrogen in modulating the relationship between the different neurotransmitter systems, mERs and the menstrual cycle. Furthering our knowledge of neurotransmitter systems involved in mental and neuropsychiatric disorders will be crucial to understanding how E2 impacts these systems and how it may be utilized to target therapeutic approaches for impairments in the serotonergic, dopaminergic, and glutamatergic systems.

## Author contributions

SZ: Writing – review & editing, Writing – original draft. PB: Writing – review & editing, Writing – original draft. AO: Writing – review & editing, Funding acquisition. PZ: Writing – review & editing, Funding acquisition. PG: Writing – review & editing, Writing – original draft, Funding acquisition, Conceptualization.

## Glossary

Abbreviation: Term
BLA: Basolateral amygdala
BLA: Cyclic adenosine 5’-monophosphate
CREB: cAMP response element-binding protein
DAT: Dopamine transporter
DOPAC: Dihydroxyphenylacetic acid
DRN: Dorsal Raphe Nucleus
DPN: Diarylpropionitrile
D2: Dopamine receptor
EB: Estradiol benzoate
ERα: Estrogen receptor alpha
ERβ: Estrogen receptor beta
ERE: Estrogen receptor element
E2: Estradiol (17β-estradiol)
GPER: G protein-coupled estrogen receptor
LTD: Long-term depression
LTP: Long-term potentiation
mERs: Membrane bound estrogen receptors
mGluRs: Metabotropic glutamate receptors
NAc: Nucleus accumbens
ORX: Orchiectomized
OVX: Ovariectomized
PFC: Prefrontal cortex
PPT: Propyl-pyrazole-triol
SERT: Serotonin reuptake transporter
SERMs: Selective estrogen receptor modulators
SSRIs: Selective serotonin reuptake inhibitors
TPH: Tryptophan hydroxylase
TPH-2: Tryptophan
5-HT: Serotonergic projections
VMN: Ventromedial hypothalamic nucleus
